# A novel risk classifier for predicting the overall survival of patients with thymic epithelial tumors based on the eighth edition of the TNM staging system: A population-based study

**DOI:** 10.3389/fendo.2022.1050364

**Published:** 2022-12-06

**Authors:** Yimeng Li, Aimin Jiang, Yujia Zhao, Chuchu Shi, Yuyan Ma, Xiao Fu, Xuan Liang, Tao Tian, Zhiping Ruan, Yu Yao

**Affiliations:** Department of Medical Oncology, The First Affiliated Hospital of Xi’an Jiaotong University, Xi’an, Shaanxi, China

**Keywords:** thymic epithelial tumors (TETs), prognostic factor, nomogram, risk classifier, TNM staging system, thymoma, thymic carcinoma, overall survival (OS)

## Abstract

**Objective:**

Thymic epithelial tumors (TETs) are rare tumors that originated from thymic epithelial cells, with limited studies investigating their prognostic factors. This study aimed to investigate the prognostic factors of TETs and develop a new risk classifier to predict their overall survival (OS).

**Methods:**

This retrospective study consisted of 1224 TETs patients registered in the Surveillance, Epidemiology, and End Results (SEER) database, and 75 patients from the First Affiliated Hospital of Xi’an Jiaotong University. The univariate and multivariate Cox regression analyses were adopted to select the best prognostic variables. A nomogram was developed to predict the OS of these patients. The discriminative and calibrated abilities of the nomogram were assessed using the receiver operating characteristics curve (ROC) and calibration curve. Decision curve analysis (DCA), net reclassification index (NRI), and integrated discrimination improvement (IDI) were adopted to assess its net clinical benefit and reclassification ability.

**Results:**

The multivariate analysis revealed that age, sex, histologic type, TNM staging, tumor grade, surgery, radiation, and tumor size were independent prognostic factors of TETs, and a nomogram was developed to predict the OS of these patients based on these variables. The time-dependent ROC curves displayed that the nomogram yielded excellent performance in predicting the 12-, 36- and 60-month OS of these patients. Calibration curves presented satisfying consistencies between the actual and predicted OS. DCA illustrated that the nomogram will bring significant net clinical benefits to these patients compared to the classic TNM staging system. The estimated NRI and IDI showed that the nomogram could significantly increase the predictive ability of 12-, 36- and 60-month OS compared to the classic TNM staging system. Consistent findings were discovered in the internal and external validation cohorts.

**Conclusion:**

The constructed nomogram is a reliable risk classifier to achieve personalized survival probability prediction of TETs, and could bring significant net clinical benefits to these patients.

## Introduction

Thymic epithelial tumors (TETs) are rare tumors that originated from thymic epithelial cells, including thymomas, thymic carcinomas, and thymic neuroendocrine neoplasms (1). According to cancer registry data, the overall incidence of thymic malignancies in the United States is 0.15 per 100,000 person-years, with higher rates among African Americans and Asian Pacific Islanders than whites or Hispanics. The incidence of TETs is slightly higher in men than in women (1.4:1) and increases with age (2). The 5-year overall survival (OS) rate is approximately 90% for thymoma and 55% for thymic carcinoma (2–4).

In 1981, Masaoka et al. developed a staging system based on whether the tumor infiltrates the envelope and surrounding tissues and organs to guide its diagnosis and treatment (5). Then Koga modified Masaoka staging based on whether it invaded the surrounding tissue (6). Up to now, the Masaoka-Koga (MK) staging system is still widely used in clinical practice. Unfortunately, although this staging system has indicated a correlation with the prognosis of thymic tumors in many studies, it is only based on a single-center small sample study over 30 years ago. Besides, the MK staging system could not fully reflect the prognostic impact of lymph node metastasis or blood metastasis from direct tumor invasion compared with the primary tumor, lymph node, and metastasis (TNM) classification of the American Joint Commission of Cancer (AJCC). Therefore, the TNM staging system has been gradually emphasized in the diagnosis and management of TETs according to the recommendations of the International Thymic Malignancy Interest Group (ITMIG) and the International Association for the Study of Lung Cancer (IASLC) in recent decades (7). Nevertheless, significant survival heterogeneity can still be observed in patients with the same TNM staging. Therefore, a more precise risk classification system should be developed to achieve personalized survival probability prediction in these patients.

Nomogram is a visual multivariate prognostic model that contains more predictors than traditional staging systems, thereby allowing individualized risk estimation. Previous publications revealed that nomogram has promising performance in predicting the survival probability of some malignancies compared to traditional TNM staging system (8, 9). To our knowledge, several nomograms were developed to predict the recurrence risk of TETs in the past few years (10–12). However, there was no relevant prognostic model constructed to predict their long-term survival probability. Herein, this study was performed to investigate the independent predictors of OS in TETs patients based on participants in the Surveillance, Epidemiology, and End Results (SEER) database, and patients from an oncology center in Northwestern China. Most importantly, we aimed to construct a reliable and personalized nomogram to predict the OS of these patients based on the eighth edition of the TNM classification system.

## Materials and methods

### Study design and participants

**Figure 1:**
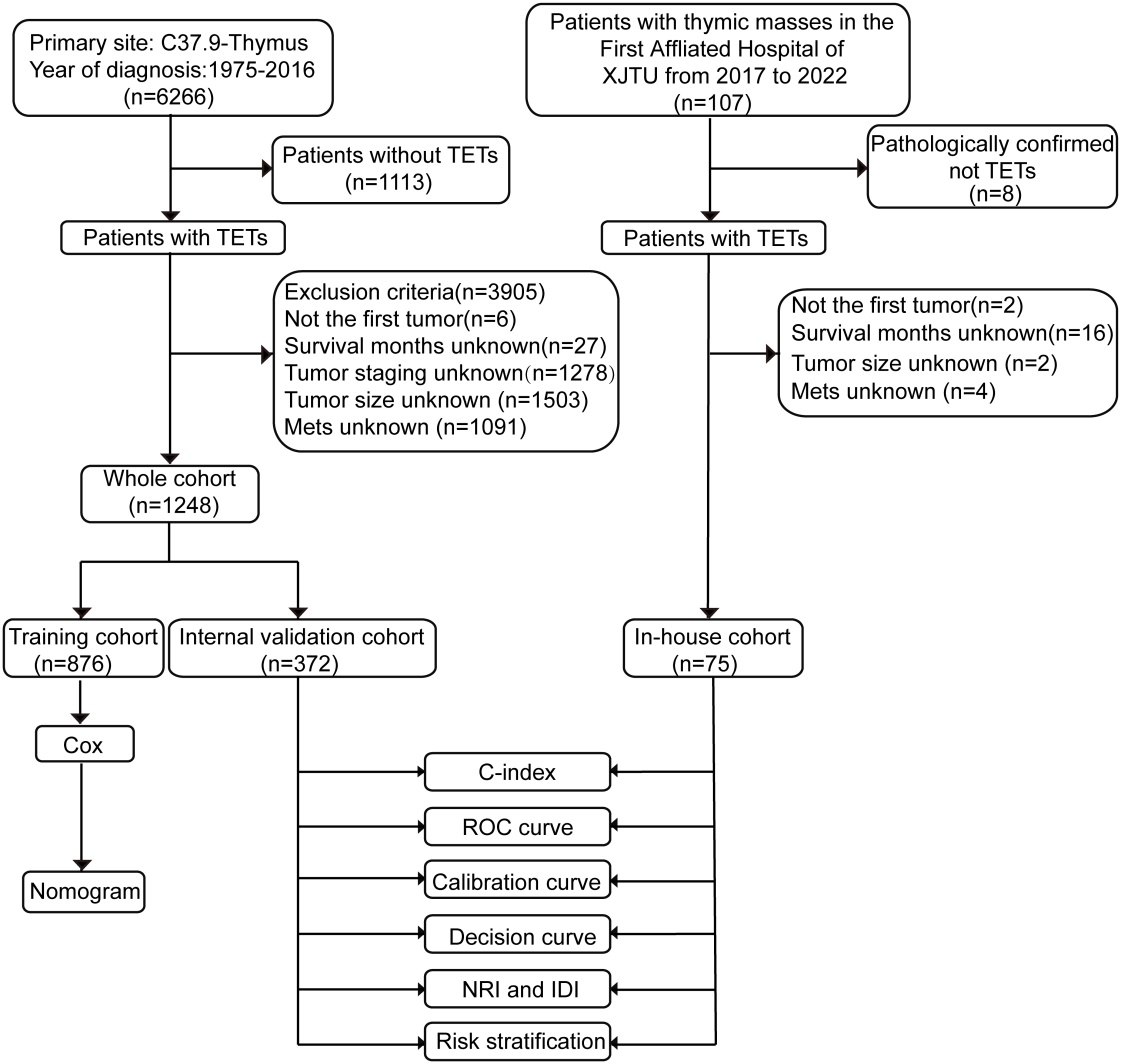
Flow chart of the study. ROC curve, receiver operating characteristics curve; NRI, net reclassification index; IDI, integrated discrimination improvement. TETs, thymic epithelial tumors; XJTU, Xi’an Jiaotong University; Mets, metastasis; C-index, the consistent index.

This is a retrospective study. Participants in this study included patients with TETs identified from the SEER database and the First Affiliated Hospital of Xi’an Jiaotong University. There are 18 cancer registries in the SEER database from the National Cancer Institute, covering nearly 30% of the U.S. population. The SEER*Stat software (version 8.3.9.2) was utilized to download patients’ information. Patients diagnosed with TETs between 1975 and 2016 from the SEER database were included in this study. First, we included the following patients according to ICD-O Morphology and Behavior Codes (1): thymoma (8580-8585, 9010); (2) thymic carcinoma (8070, 8123, 8082, 8140, 8260, 8200, 8144, 8560, 8023, 8430, 8310, 8033 8980, 8020, 8586); (3) thymic neuroendocrine tumors (8240, 8249, 8041, 8045, 8013) (1). TETs were not the first tumor, TNM or MK stage could not be calculated, or patients without fully documented survival time, tumor size, and metastasis records were excluded. Finally, a total of 1248 patients were enrolled from the SEER database. Besides, 75 patients diagnosed with TETs with complete medical records from the First Affiliated Hospital of Xi’an Jiaotong University were included in this study. This study was approved by the ethics committee of the First Affiliated Hospital of Xi’an Jiaotong University. The flow chart for patient selection and nomogram construction and validation is shown in **Figure 1**. We conducted this study following the requirements of the Declaration of Helsinki.

### Cohort establishment and variable selection

Patients in the SEER database were randomly divided into a training cohort and an internal validation cohort in a 7:3 ratio through the “createDataPartition” function in the R software. The training cohort is used to identify the independent predictors of OS and construct the nomogram to predict their survival probability. The internal validation cohort is utilized to validate its predictive ability. Besides, seventy-five patients from our medical center served as an independent external validation cohort to validate the generality of the nomogram. Eighteen common covariables both in the SEER database and our in-house cohort were collected, including age, sex, race, marital status, histologic type, tumor grade, tumor size, distant metastases sites (bone, brain, liver, and lung), local infiltrates, regional lymph nodes involved, regional nodes positive, distant lymph nodes involved, and treatment modalities (surgery, radiation, and chemotherapy). We referred to local infiltrates, lymph nodes involved, and metastasis to work out the 8^th^ edition of AJCC-TNM staging and MK staging of each patient. Age and tumor size were transformed into categorical variables by setting 65 years as the cut-off value of age and 5.5 cm as the cut-off value of tumor size (13). Then, the single-factor and multi-factor Cox regression analyses were performed to identify the independent prognostic variables of OS and thereby develop the nomogram.

### Statistical analysis

All categorical variables were presented as frequency and percentage, and the chi-square test or fisher exact test was utilized to compare the differences between different groups. Kaplan-Meier survival curve was generated to compare the survival difference between different groups, with a log-rank test being adopted to test the statistical significance. The univariate and multivariate Cox regression analyses were applied to identify independent prognostic factors of OS for patients with TETs. Variables with a *P* value <0.1 in the univariate Cox regression analysis were then incorporated into the multivariate Cox regression analysis. The hazard ratio (HR) and 95% confidence interval (CI) of each variable were estimated to investigate their associations with the OS of patients with TETs. The significant variables in the multivariable Cox regression analysis were selected for nomogram construction, with R software, “rms” and “regplot” packages being employed to visualize the nomogram. In addition, the consistent index (C-index), receiver operating characteristic (ROC) curve, and calibration curve were used to assess the discrimination and calibration abilities of the nomogram in each cohort. Furthermore, decision curve analysis (DCA) was adopted to compare the net clinical benefits of the nomogram and the traditional 8^th^ edition of the AJCC-TNM staging system when they were adopted to guide clinical decision-making. Ultimately, the net reclassification index (NRI) and the integrated discrimination index (IDI) were calculated to evaluate the reclassification ability of the nomogram compared to the traditional 8^th^ edition of the AJCC-TNM staging system. The statistical difference was considered as significant when *P*<0.05. All tests were two-sided. All statistical analyses and visualization were achieved *via* R software (version 4.1.2) and Jamovi software (version 1.6.23) for Windows 64.0.

## Results

### Clinical characteristics of the participants

The average age of patients in the whole SEER database was 60.2 years old, which involved 658 males and 590 females. Thymoma was the most prevalent histologic type, accounting for 71.8% of cases. There were 72.8% of TETs patients diagnosed with TNM stage I-III. Regarding the detailed therapeutic regimens, 80.5% of patients received surgery, 33.3% received chemotherapy, and 46.5% received radiation. Besides, we observed that the lung was the most common distant metastatic organ, accounting for 8.01% of patients, followed by bone (2.64%) and liver (1.60%). Patients in the SEER database were randomly divided into the training and internal validation cohort in a ratio of 7:3, and there was no significant difference in baseline characteristics between these cohorts (**Table 1**, all *P*- value >0.05). In our cohort, a total of 75 patients with TETs were identified from 2017 to 2022. More patients suffered from liver metastases in our cohort compared to the SEER database. Most clinicopathological parameters were comparable between the SEER database and our in-house cohort. **Table 1** detailed summarized the clinical characteristics of patients with TETs enrolled in this study.

**Table 1:** Demographic and clinical characteristics of patients with TETs.

Characteristics		Whole population(n = 1248)	Training cohort(n = 876)	Internal validation cohort (n = 372)	*P* ^a^	In-house Cohort (n = 75)	*P* ^b^
**Sex (n, %)**	Male	658 (52.7)	457 (52.2)	201 (54.0)	0.953	39 (52.0)	1.000
	Female	590 (47.3)	419 (47.8)	171 (46.0)		36 (48.0)	
**Age (n, %)**	<65 years old	734 (58.8)	517 (59.0)	217 (58.3)	0.692	53 (70.7)	0.064
	≥65 years old	514 (41.2)	359 (41.0)	155 (41.7)		22 (29.3)	
**Race (n, %)**	White	835 (66.9)	593 (67.7)	242 (65.1)	0.785	0 (0)	<0.001^*^
	Black	180 (14.4)	116 (13.2)	64 (17.2)		0 (0)	
	Others	233 (18.7)	167 (19.1)	66 (17.7)		75 (100)	
**Marital status (n, %)**	Married	750 (60.1)	518 (59.1)	232 (62.4)	0.158	72 (96.0)	<0.001^*^
	Others	498 (39.9)	358 (40.9)	140 (37.6)		3 (4.0)	
**Histologic type (n, %)**	Thymoma	896 (71.8)	629 (71.8)	267 (71.8)	0.121	50 (66.7)	0.611
	Thymic carcinoma	301 (24.1)	211 (24.1)	90 (24.2)		22 (29.3)	
	Thymic neuroendocrine neoplasms	51 (4.09)	36 (4.1)	15 (4.0)		3 (4.0)	
**Grade (n, %)**	I-II	111 (8.89)	82 (9.4)	29 (7.8)	0.567	3 (4.0)	0.071
	III-IV	152 (12.2)	109 (12.4)	43 (11.6)		15 (20.0)	
	Unknown	985 (78.9)	685 (78.2)	300 (80.6)		57 (76.0)	
**T stage (n, %)**	T0-T1a	499 (40.0)	350 (40.0)	149 (40.1)	0.772	39 (52.0)	0.056
	T1b-T4	749 (60.0)	526 (60.0)	223 (59.9)		36 (48.0)	
**N stage (n, %)**	N0	1072 (85.9)	753 (86.0)	319 (85.8)	0.798	67 (89.3)	0.523
	N1-2	176 (14.1)	123 (14.0)	53 (14.2)		8 (10.7)	
**M stage (n, %)**	M0	996 (79.8)	700 (79.9)	296 (79.6)	0.242	60 (80.0)	1.000
	M1a-M1b	252 (20.2)	176 (20.1)	76 (20.4)		15 (20.0)	
**TNM stage (n, %)**	I-III	908 (72.8)	634 (72.4)	274 (73.7)	0.448	57 (76.0)	0.588
	IV	340 (27.2)	242 (27.6)	98 (26.3)		18 (24.0)	
**Masaoka-Koga stage (n, %)**	I-IIA	436 (34.9)	308 (35.2)	128 (34.4)	0.326	31 (41.3)	0.551
	IIB-III	472 (37.8)	326 (37.2)	146 (39.2)		26 (34.7)	
	IV	340 (27.2)	242 (27.6)	98 (26.3)		18 (24.0)	
**Tumor size (n, %)**	<5.5cm	434 (34.8)	316 (36.1)	118 (31.7)	0.953	35 (46.7)	0.089
	≥5.5cm	814 (65.2)	560 (63.9)	254 (68.3)		40 (53.3)	
**Surgery (n, %)**	None/Unknown	243 (19.5)	181 (20.7)	62 (16.7)	0.692	20 (26.7)	0.282
	Yes	1005 (80.5)	695 (79.3)	310 (83.3)		55 (73.3)	
**Radiation (n, %)**	None/Unknown	668 (53.5)	474 (54.1)	194 (52.2)	0.785	43 (57.3)	0.677
	Yes	580 (46.5)	402 (45.9)	178 (47.8)		32 (42.7)	
**Chemotherapy (n, %)**	None/Unknown	833 (66.7)	582 (66.4)	251 (67.5)	0.158	52 (69.3)	0.702
	Yes	415 (33.3)	294 (33.6)	121 (32.5)		23 (30.7)	
**Bone metastasis (n, %)**	None	1215 (97.4)	854 (97.5)	361 (97.0)	0.158	72 (96.0)	0.440
	Yes	33 (2.64)	22 (2.5)	11 (3.0)		3 (4.0)	
**Brain metastasis (n, %)**	None	1241 (99.4)	873 (99.7)	368 (98.9)	0.567	74 (98.7)	0.280
	Yes	7 (0.56)	3 (0.3)	4 (1.1)		1 (1.33)	
**Liver metastasis (n, %)**	None	1228 (98.4)	864 (98.6)	364 (97.8)	0.772	71(94.7)	0.031
	Yes	20 (1.60)	12 (1.4)	8 (2.2)		4(5.33)	
**Lung metastasis (n, %)**	None	1148 (92.0)	801 (91.4)	347 (93.3)	0.798	64(85.3)	0.119
	Yes	100 (8.01%)	75 (8.6)	25 (6.7)		11(14.7)	

TETs, thymic epithelial tumors.

*P^a^, P* value between training cohort and validation; *P^b^, P* value between training cohort and in-house cohort.

### Univariate and multivariate Cox regression analysis

We performed a single-factor Cox regression analysis in the training cohort to identify potential prognostic factors of OS in patients with TETs. The results showed that age, sex, marital status, histologic type, TNM staging, Masaoka-Koga staging, tumor grade, tumor size, bone metastasis, brain metastasis, liver metastasis, lung metastasis, surgery, radiation, and chemotherapy were associated with the OS of these patients (all *P*-value <0.05). Next, we incorporated the above variables into a multivariate Cox regression analysis to minimize the impact of confounders. Furthermore, considering the correlation between the TNM staging system and the MK staging system, we only selected the former for the regression equation. Ultimately, the results indicated that age, sex, histologic type, TNM staging, tumor grade, tumor size, surgery, and radiation were the independent prognostic factors of OS for patients with TETs (**Table 2**). Among them, age ≥ 65 years old (HR: 2.01, 95%CI: 1.50-2.71), thymic carcinoma (HR: 1.81, 95%CI: 1.29-2.55), grade III-IV (HR: 2.50, 95%CI: 1.31-4.79), tumor size ≥ 5.5 cm (HR: 1.48, 95%CI: 1.07-2.05), and TNM stage IV (HR: 2.29, 95%CI: 1.59-3.31) were significantly correlated to the unfavorable OS of patients with TETs. On the contrary, female patients (HR: 0.65, 95%CI: 0.48-0.89) and patients who received surgery (HR: 0.35, 95%CI: 0.26-0.49) and radiation (HR: 0.65, 95%CI: 0.49-0.87) had longer OS (**Table 2**).

**Table 2:** Univariate and multivariate Cox analyses on variables for the prediction of OS of patients with TETs.

Characteristics	Univariate analysis			Multivariate analysis		
	HR	95% CI	*P* value	HR	95%CI	*P* value
Sex (Female vs. Male)	0.76	0.58-1.00	0.054	0.66	0.49-0.88	0.005*
Age (years, ≥65 vs. <65)	2.07	1.57-2.73	<0.001	1.94	1.46-2.59	<0.001*
Marital status (Others vs. Yes)	1.27	0.96-1.67	0.091	1.32	0.99-1.76	0.058
Thymoma	Reference			Reference		
Thymic carcinoma	2.74	2.06-3.62	<0.001	1.81	1.29-2.55	0.001*
Thymic neuroendocrine neoplasms	0.50	0.16-1.59	0.243	0.36	0.11-1.19	0.093
Grade						
I-II	Reference			Reference		
III-IV	3.68	1.95-6.93	<0.001	2.50	1.31-4.79	0.006*
Unknown	1.47	0.82-2.65	0.200	1.68	0.91-3.11	0.096
Tumor size (≥5.5cm vs. <5.5cm)	1.63	1.19-2.23	0.002	1.48	1.07-2.05,	0.018*
TNM stage (IV vs. I-III)	3.64	2.76-4.79	<0.001	2.29	1.59-3.31	<0.001*
Surgery (Yes vs. None/Unknown)	0.22	0.16-0.28	<0.001	0.35	0.26-0.49	<0.001*
Radiation (Yes vs. None/Unknown)	0.78	0.59-1.03	0.083	0.65	0.49-0.87	0.004*
Chemotherapy (Yes vs. None/Unknown)	2.19	1.66-2.88	<0.001	0.86	0.61-1.22	0.396
Bone metastasis (Yes vs. None)	3.49	1.99-6.12	<0.001	1.26	0.64-2.49	0.497
Brain metastasis (Yes vs. None)	7.21	1.78-29.24	0.006	1.73	0.41-7.36	0.459
Liver metastasis (Yes vs. None)	3.69	1.73-7.85	0.001	0.93	0.40-2.16	0.872
Lung metastasis (Yes vs. None)	2.51	1.73-3.65	<0.001	0.89	0.57-1.40	0.625

OS, overall survival; TETs, thymic epithelial tumors; HR, hazard ratio; CI, confidence interval.

*represents *P* value< 0.05.

### Nomogram development and validation

**Figure 2:**
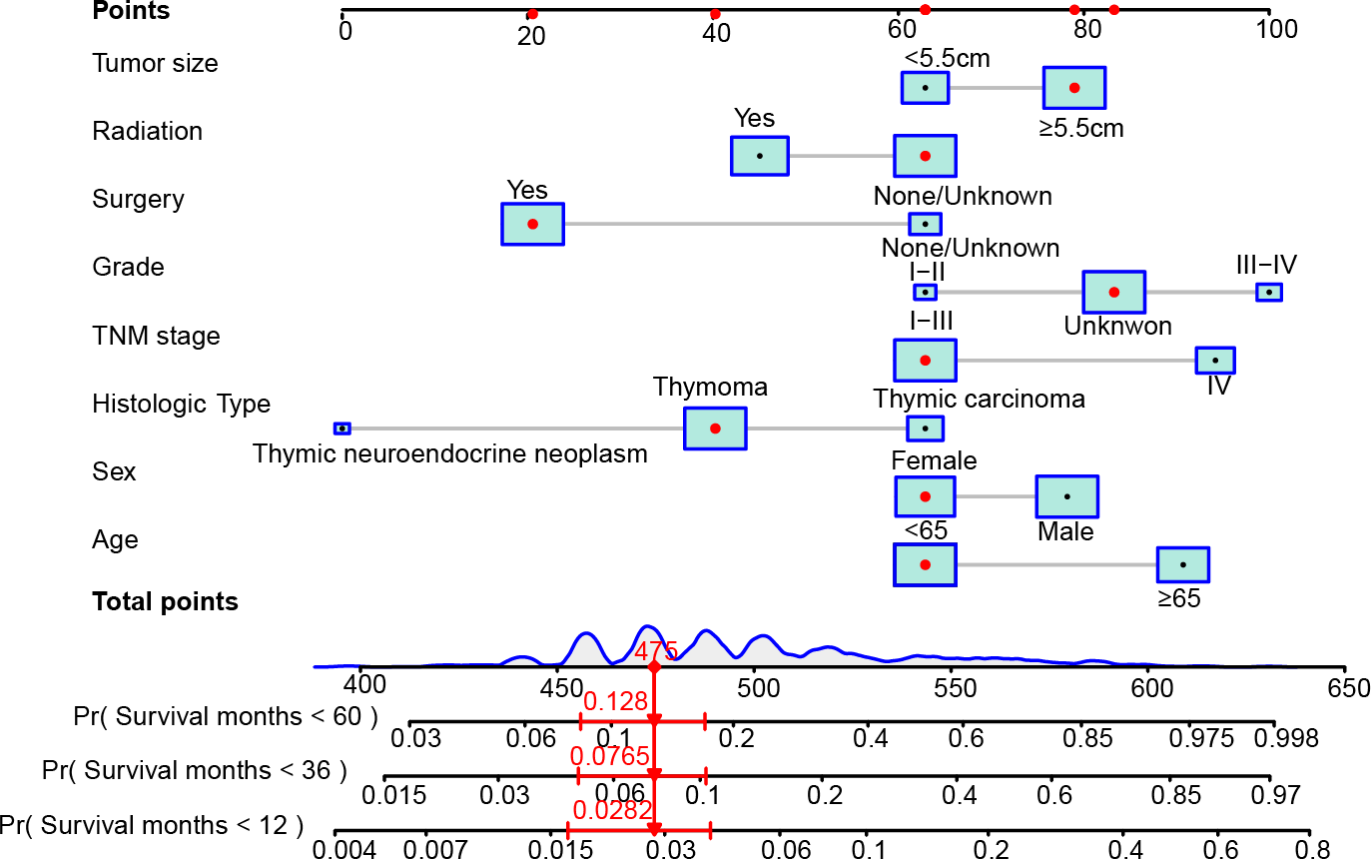
The constructed nomogram to predict the 12-, 36-, and 60-month OS of patients with TETs according to the eight independent prognostic factors identified in the multivariate Cox regression analysis. In the nomogram, the patient was a 66 years old female, diagnosed with stage IV (T_4_N_0_M_1b_) thymoma after surgery. The tumor size of this patient excessed 5.5 cm and the differentiation of the tumor was unknown. This patient did not receive radiotherapy after surgery. According to the nomogram, the total points of this patient is 475, and the probability of OS less than 12-, 36-, and 60-month of this patient is 2.82%, 7.65%, and 12.8%, respectively.

**Figure 3:**
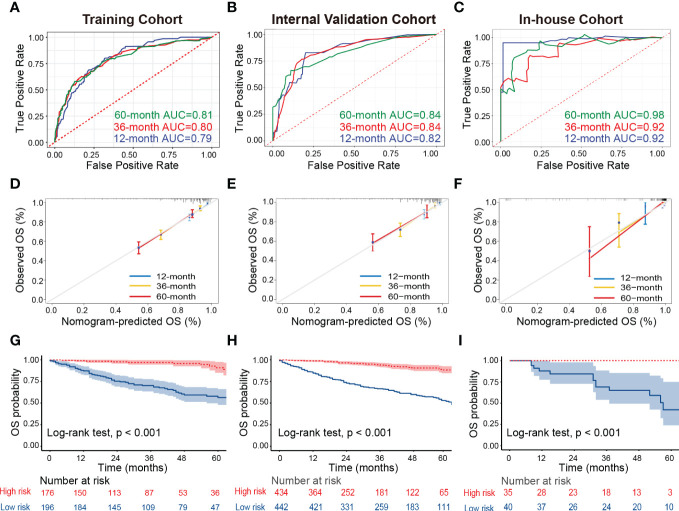
Assessment of the discrimination and calibration abilities of the constructed nomogram. **(A–C)** The ROC curves for predicting 12-, 36-, and 60-month OS of patients with TETs in the training cohort **(A)**, internal validation cohort **(B)**, and in-house cohort **(C)** based on the nomogram. **(D–F)** The calibration curves for predicting 12-, 36-, and 60-month OS of patients with TETs in the training cohort **(D)**, internal validation cohort **(E)**, and in-house cohort **(F)** are based on the nomogram. **(G–I)** Kaplan-Meier survival curves to display the risk stratification ability of the nomogram in the training cohort **(G)**, internal validation cohort **(H)**, and in-house cohort **(I)**. ROC, receiver operating characteristic curves; TETs, thymic epithelial tumors; OS, overall survival.

Then, we developed the nomogram based on the eight independent prognostic factors of OS in patients with TETs to achieve personalized survival probability prediction. As vividly illustrated in **Figure 2**, clinicians could easily predict the 12-, 36-, and 60-month OS probability of each patient according to the constructed nomogram. The ROC curves revealed that the nomogram had a remarkable discrimination ability in predicting the 12- (AUC: 0.79), 36- (AUC: 0.80), and 60- (AUC: 0.81) month OS of TETs in the training cohort (**Figure 3A**). Similar results were also observed in the internal and external validation cohorts (**Figures 3B, C**). Besides, the estimated C-index also demonstrated that the constructed nomogram had excellent discrimination power in predicting the OS of these patients (training cohort: 0.781, internal validation cohort: 0.828, and external validation cohort: 0.911; respectively). Furthermore, we also generated calibration curves to evaluate the calibration ability of the nomogram, which also illustrated higher consistencies between the actual and predicted OS in all the cohorts (**Figures 3D–F**). To conclude, the constructed nomogram had a promising performance in predicting the survival probability of patients with TETs.

### Risk stratification ability assessment of the nomogram

Ultimately, all patients with TETs were divided into low-risk and high-risk groups based on the median of the total points derived from the nomogram to assess the risk stratification ability of the nomogram in each cohort. Kaplan-Meier survival curves displayed that the OS of patients with TETs in the high-risk group was significantly decreased than those in the low-risk group in the training cohort (**Figure 3G**), internal validation cohort (**Figure 3H**), and our in-house cohort (**Figure 3I**). These results supported that the constructed nomogram had excellent risk stratification ability.

### Clinical utility evaluation of the nomogram

**Figure 4:**
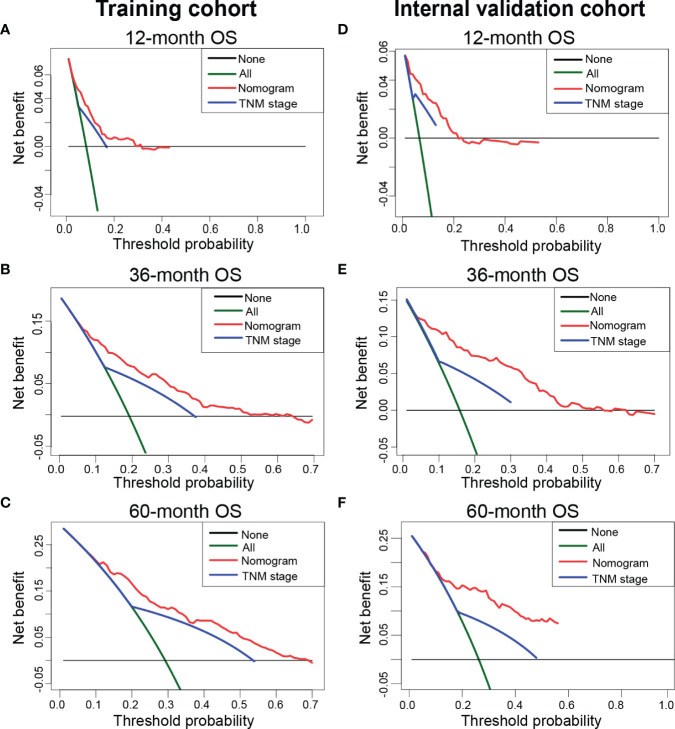
Decision curve analysis to compare the net clinical benefits of the nomogram and classic TNM staging system when they were adopted to guide clinical practice. **(A–C)** The net clinical benefits of 12- **(A)**, 36- **(B)**, and 60-month **(C)** OS in the training cohort. **(D-F)** The net clinical benefits of 12- **(D)**, 36- **(E)**, and 60-month **(F)** in the internal validation cohort. OS, overall survival.

We applied DCA to compare the net clinical benefits of patients with TETs when the nomogram and 8^th^ edition of the TNM staging system were adopted to guide the clinical practice. We observed that the nomogram could bring more net clinical benefits to these patients compared with the TNM staging system in predicting the 12-, 36-, and 60-month OS at specific risk thresholds in the training cohort (**Figures 4A–C**), internal validation cohort (**Figures 4D–F**), and external validation cohort (**Supplementary Figure 1**). Furthermore, NRI and IDI were estimated to compare the reclassification abilities of the nomogram and the 8^th^ edition of the TNM staging system. The estimated NRI illustrated that the nomogram could significantly improve the predictive accuracy rate in predicting the 12- (27.9%), 36- (37.2%), and 60- (7.1%) month OS probability of patients with TETs compared to the TNM staging system (**Table 3**). The constructed nomogram also provided a significant improvement of IDI in predicting the 12- (5.3%), 36- (9.7%), and 60- (10.0%) month OS probability of patients with TETs compared to the TNM staging system (**Table 3**). Consistent results were observed in the internal validation cohort. In our in-house cohort, the constructed nomogram only provided significant improvement of NRI and IDI in predicting the 12-month OS probability of patients with TETs (**Table 3**).

**Table 3:** NRI and IDI of the nomogram versus the TNM staging system for predicting OS of patients with TETs.

Index	Training cohort					Validation cohort						In-house Cohort		
	Estimate		95% CI	*P* value		Estimate		95% CI	*P* value			Estimate	95% CI	*P* value
NRI (vs. the TNM staging system)														
For 1-year survival	0.279	0.345-0.392				0.362	0.045-0.605					0.603	-0.014-1.199	
For 3-year survival For 5-year survival	0.372 0.071	-0.021-0.473 -0.049-0.556				0.466 0.148	0.149-0.698 0.010-0.786					-0.111 -0.149	-0.162-1.204 -0.256-0.254	
IDI (vs. the TNM staging system)														
For 1-year survival	0.053	0.030-0.096		<0.001		0.081	0.040-0.187		<0.001			0.324	-0.070-0.622	0.080
For 3-year survival For 5-year survival	0.097 0.100	0.064-0.149 0.062-0.164		<0.001 <0.001		0.161 0.241	0.102-0.277 0.163-0.352		<0.001 <0.001			0.139 0.038	0.016-0.419 -0.247-0.375	0.038 0.591

NRI, net reclassification index; IDI, discrimination improvement; OS, overall survival, CI, confidence interval; TETs, thymic epithelial tumors.

### The impact of treatment modalities on different histologic subtypes

**Figure 5:**
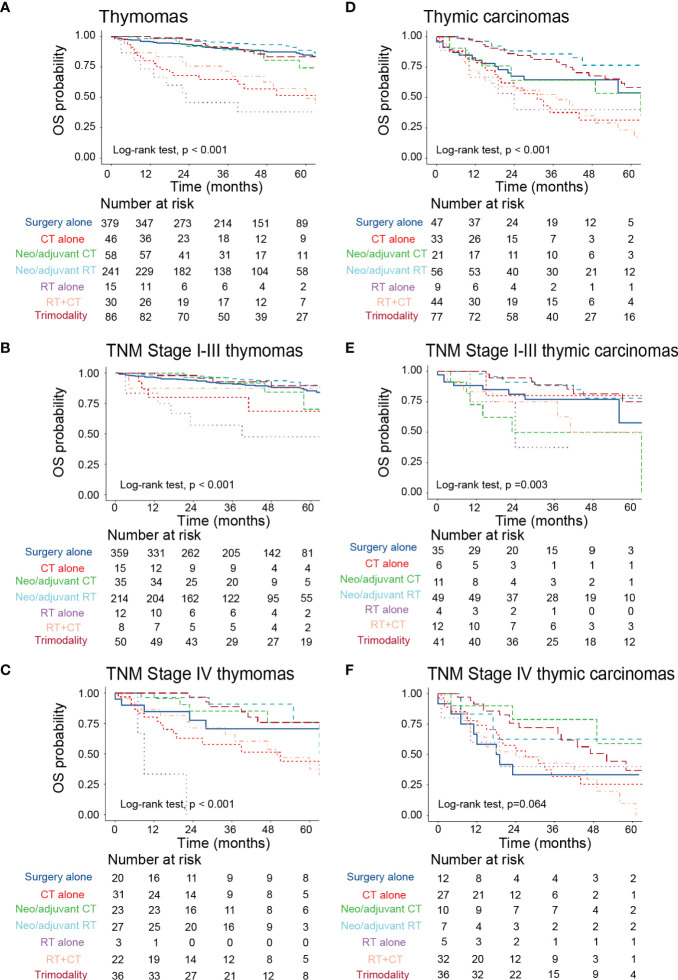
The impact of different treatment modalities on the prognosis of patients with TETs with different histologic subtypes. **(A–C)** Kaplan-Meier survival curves to display the survival difference among all stage thymomas **(A)**, TNM stage I-III thymomas **(B)**, and TNM stage IV thymomas **(C)** who received different treatments. **(D–F)** Kaplan-Meier survival curves to display the survival difference among all stage thymic carcinomas **(D)**, TNM stage I-III thymic carcinomas **(E)**, and TNM stage IV thymic carcinomas **(F)** who received different treatments. TETs, thymic epithelial tumors; RT, radiotherapy; CT, chemotherapy; OS, overall survival.

Then, we explored the impact of different therapeutic regimens on the prognosis of patients with thymoma and thymic carcinoma. In the thymoma subgroup, neoadjuvant/adjuvant radiation, surgery alone, trimodality therapy, and neoadjuvant/adjuvant chemotherapy could improve patients’ OS compared to chemoradiation, radiation alone, and chemotherapy alone (**Figure 5A**). The efficacy of the above therapies was similar for patients with TNM stage I-III thymoma (**Figure 5B**). However, for TNM stage IV thymoma, patients who only received radiation were associated with the worst survival probability (**Figure 5C**). Among the thymic carcinomas, neoadjuvant/adjuvant radiation and trimodality therapy provided more survival benefits to these patients compared to surgery alone, chemotherapy alone, radiation alone, chemoradiation, and neoadjuvant/adjuvant chemotherapy (**Figure 5D**). Similar results were observed in patients with TNM stage I-III thymic carcinoma (**Figure 5E**). In the TNM stage IV thymic carcinoma subgroup, there was no statistical difference in survival probability between different treatment groups (**Figure 5F**).

## Discussion

In the current study, we investigated the independent prognostic factors of TETs based on patients from the SEER database and our cohort. Moreover, a novel nomogram was developed to predict their OS, with excellent performance observed in the training cohort, internal validation cohort, and external validation cohort. The multivariate Cox regression analysis identified that age, sex, histologic type, TNM staging, tumor grade, tumor size, surgery, and radiation were independent predictors of OS in patients with TETs. This is in general agreement with previously published studies (13–15). The impact of tumor size on the prognosis of patients with TETs remains controversial. For instance, in 2014, the IASLC/ITMIG reported that tumor size was not correlated to the clinical outcome of patients with TETs (16). However, Yun et al. indicated that tumor size was an independent predictor of OS and recurrence-free survival in patients with completely resected limited-stage TETs in a real-world multicenter study conducted in Korea (13). Besides, they revealed that the optimal cutoff value for tumor size was >5.5 cm for both OS and recurrence-free survival in these patients (13). Consistent findings were reported by Khorfan and colleagues (17, 18). The heterogeneity between different studies could explain this discrepancy (16). Thus, multicenter prospective studies are urgently needed to investigate the role of tumor size on the prognosis of patients with TETs in the future. In 2014, the IASLC/ITMIG emphasized the role of the 8^th^ edition of the TNM staging system in the diagnosis and management of TETs (7). Recently, several studies also explored the prognostic significance of the TNM staging system in patients with TETs (19, 20). Consistent with our results, Tian et al. reported that the TNM staging system was an independent predictor of OS in patients with TETs (19, 20). Therefore, the TNM staging system should be considered in clinical practice when clinicians determined the staging and treatment options of these patients. Meanwhile, multicenter prospective studies could be designed to compare the performance of the TNM and the MK systems in predicting the prognosis of patients with TETs. Numerous studies have found that complete resection was the most important factor influencing the prognosis of TETs (17, 21, 22). Besides, a meta-analysis also elucidated that advanced unresectable thymomas can still benefit from tumor reduction surgery (23). In the current study, we identified that radiation was correlated with favorable OS in patients with TETs. However, the role of postoperative radiation in thymoma and thymic carcinoma remains controversial (22, 24, 25). Until 2017, Jackson and colleagues systematically investigated the role of postoperative radiation in thymoma and thymic carcinoma (25). They observed that postoperative radiation was significantly associated with improved OS in these patients (25). Subgroup analysis indicated that postoperative radiation could provide the greatest relative benefits for MK stage IIB to III disease and positive margins (25).

Nowadays, the nomogram is a widely used predictive tool to predict the survival probability of cancer patients (8, 9). It could easily visualize the risk of each patient according to the contribution to the study outcome of variables in the multivariate analysis. Here, we developed a nomogram based on the above eight variables to predict the survival probability of patients with TETs at 12-, 36-, and 60-month. Subsequent ROC curves and calibration curves demonstrated that the constructed nomogram yielded acceptable discrimination ability and calibration ability in predicting the 12-, 36-, and 60-month OS of these individuals. As we all know, ROC curves and calibration curves are based on the sensitivity and specificity of the model, and thus could not reflect “false positive” and “false negative” cases. Hence, DCA was developed to fill this gap in evaluating the performance of the predictive model by considering the net clinical benefit (26). The traditional TNM staging system was a commonly recognized risk stratification tool in cancer diagnosis and management. Numerous studies have identified that TNM staging is significantly correlated with the prognosis of patients with solid tumors (27). How about the performance of the constructed nomogram in predicting the survival probability of patients with TETs compared to the TNM staging system? The results indicated that our nomogram will bring significant net clinical benefit improvement of these patients when it was adopted to support clinical decision-making compared to the classic 8^th^ edition of the TNM staging system. Meanwhile, IDI and NRI also supported that our nomogram could significantly improve the reclassification accuracy rate compared to the classic 8^th^ edition of the TNM staging system. Together, the constructed nomogram has promising performance in predicting the 12-, 36-, and 60-month OS of patients with TETs.

Then, we explored the impact of different therapeutic regimens on the prognosis of patients with thymoma and thymic carcinoma. We identified that neoadjuvant/adjuvant radiation and trimodality therapy could provide significant survival benefits to patients with thymoma and thymic carcinoma. Subgroup analysis stratified by TNM staging demonstrated that neoadjuvant/adjuvant radiation, surgery alone, trimodality therapy, and neoadjuvant/adjuvant chemotherapy provided potential survival benefits for TNM stage I-III thymoma. However, patients with TNM stage IV thymoma could not benefit from radiotherapy. Consistent results were obtained in a recently published study by Khorfan and colleagues (17). They indicated that MK stage IV (TNM stage IV) thymoma solely treated with radiation or chemotherapy had a relatively lower 5-year survival probability compared to other surgical-containing therapies (17). Some studies showed that subtotal resection improved the prognosis of patients with advanced unresectable thymoma (21, 23). Therefore, multimodal treatment including systemic therapy should be considered for metastatic unresectable thymoma (28, 29). There is limited evidence guiding the management of patients with advanced-stage thymoma until now. Khorfan et al. indicated that surgical resection followed by adjuvant radiation was associated with the longest survival for both TNM stage III and IV thymoma (17). Besides, they found that induction therapy was not correlated to the completeness of resection (17). However, further prospective studies could be designed to appropriately evaluate its role in the treatment of advanced-stage thymoma. Among the thymic carcinomas, neoadjuvant/adjuvant radiation and trimodality therapy provided more survival benefits to TNM stage I-III thymic carcinoma. However, the above treatment modalities did not show significant survival differences in TNM stage IV thymic carcinoma. Recently, ITMIG updated the advances in the management of thymic carcinoma (30). Surgical resection maintains a central role in the management of early-stage and locally advanced thymic carcinomas (30). Well-planned resection in combination with radiotherapy could also bring significant survival benefits for locally advanced tumors including those with oligometastatic pleural and or pericardial disease (30). Patients with positive margins and patients with completely resected MK stage II to IVA (TNM stage T_1b_–T_4_, N_0–2_, M0–1a) diseases should be strongly considered for adjuvant radiation therapy (22, 30). However, treatment available for advanced unresectable thymic carcinoma is limited due to its rarity (31–33). Platinum-based combination chemotherapy is used for the frontline treatment of metastatic thymic carcinomas due to its aggressive behaviors (34). However, the efficacy of chemotherapy may be limited due to the more aggressive character of metastatic thymic carcinoma (32, 35, 36). In the study conducted by Ryo et al, patients with MK stage IVB thymic carcinoma who had received chemotherapy benefited from volume reduction surgery in terms of OS (35). Ye and Yusuke et al. investigated that surgical treatment may be beneficial for OS in MK stage IV thymic carcinoma patients (37, 38). Furthermore, Xue et al. reported that surgical resection with post-chemotherapy radiotherapy provided a progression-free survival(PFS) benefit for MK stage IV patients receiving first-line chemotherapy (32). However, considering the complexity of this disease, multidisciplinary team decisions should be considered during the management of thymic carcinoma, especially advanced thymic carcinoma (39–42).

Despite the advantages of our study, there are still some inevitable limitations in this study. First, selection bias and informative bias could not be avoided due to the retrospective design of this study. Second, although the SEER database provided a large sample size of patients, some important variables are still unavailable. For instance, the detailed chemotherapeutic regimens and lines, the concrete dosage and types of radiation, and the detailed surgical types. Third, in recent decades, immunotherapy, anti-angiogenic therapy, and targeted therapy are increasingly used in TETs patients and could provide a significant survival benefit to these patients. However, we could not further investigate the role of these treatments on the prognosis of patients with TETs since these variables were not recorded in the database. Hence, well-designed, multicenter, large-scale, and prospective studies should be conducted in the future to provide more profound insights into this field.

## Conclusions

To conclude, we identified eight independent predictors of OS in patients with TETs and constructed a nomogram that could effectively predict the OS probability of these individuals. The constructed nomogram could bring significant net clinical benefits to these patients compared to the classic 8^th^ edition of the TNM staging system. The performance of the nomogram was validated in the internal validation cohort and our medical center. However, large-scale prospective studies are still urgently needed to validate our findings.

## Data availability statement

The datasets presented in this study can be found in online repositories. The names of the repository/repositories and accession number(s) can be found below: https://seer.cancer.gov/.

## Author contributions

Conception/design: YY, ZR, XL, TT, and XF. Provision of study material: YL, AJ, YZ, CS, and YM; Collection and/or assembly of data: YL, AJ, and YZ; Data analysis and interpretation: YL, AJ, CS, and YM; Manuscript writing: YL and AJ; Final approval of manuscript: YY and ZR. All authors read and approved the final manuscript and agree to be accountable for all aspects of the research in ensuring that the accuracy or integrity of any part of the work is appropriately investigated and resolved.

## Supplementary material

Supplementary Figure 1 Decision curve analysis of the nomogram for predicting the net benefit rates of patients with TETs. **(A–C)** The net benefit rates of 12- **(A)**, 36- **(B)**, and 60-month **(C)** in the in-house cohort. TETs, thymic epithelial tumors; OS, overall survival.
