# Supplementary figures and images for: A novel risk classifier for predicting the overall survival of patients with thymic epithelial tumors based on the eighth edition of the TNM staging system: A population-based study
Source: Front Endocrinol (Lausanne). 2022 Dec 6;13:1050364. doi: 10.3389/fendo.2022.1050364 (PMC9763871; doi:10.3389/fendo.2022.1050364)

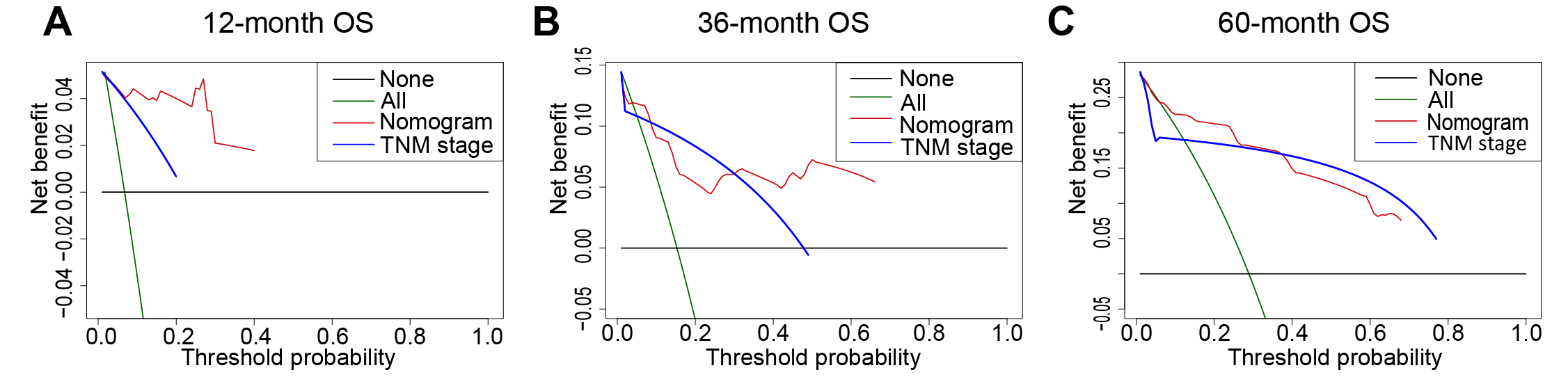

Supplement: Supplementary Figure 1 — Decision curve analysis of the nomogram for predicting the net benefit rates of patients with TETs. (A–C) The net benefit rates of 12- (A), 36- (B), and 60-month (C) in the in-house cohort. TETs, thymic epithelial tumors; OS, overall survival. [file Image_1.tif]
